# News and Views

**DOI:** 10.1007/s43673-021-00024-1

**Published:** 2021-11-09

**Authors:** AAPPS

**Affiliations:** Association of Asia Pacific Physical Societies, Seoul, South Korea

## Tailoring Topological Phases in Two-dimensional Transition Metal Dichalcogenides by Feng-Chuan Chuang

Recently, topological materials and two-dimensional materials have attracted a significant amount of research interest in condensed matter physics. In particular, topological insulators (TIs) have been intensively studied for the past decade due to their interesting electrical, optical, and mechanical properties. In addition, two-dimensional topological insulators (2D TIs), also known as quantum spin Hall (QSH) insulators, exhibit unique symmetry-protected helical metallic edge states with an insulating interior, making these materials well-suited for optoelectronics, spintronics, quantum computing, and other applications due to the robustness of their edge states against backscattering. A distinct characteristic of 2D TIs is the band inversion between the valence band maximum (VBM) and conduction band minimum (CBM) at the Fermi level. Since the electronic properties of materials can be engineered by adding or removing an electron or a hole, the location of the Fermi level can be tuned in such a way that it coincides with the band inversion.

The computational materials research group in the Department of Physics, National Sun Yat-sen University led by Dr. Feng-Chuan Chuang has aggressively studied the fascinating research topic on 2D topological materials. One of the driving factors is that most of the previously found 2D TIs exhibited small band gaps which are not suitable for room-temperature applications, thus, the campaign to search for large band gap 2D TIs began. Another factor is that most of these free-standing 2D materials are highly sensitive to the supporting substrate resulting in the vanishment of topological phases. These factors inspired Chuang’s group to find promising material alternatives with larger band gaps and suitable substrates.

One of the most successful material designs of large-bandgap 2D TIs by Chuang’s group is the case of planar bismuthene grown on SiC(0001) in 2015 [1]. Bismuthene adapted a similar honeycomb-like structure with a stronger spin-orbit interaction. However, no experimental group had successfully synthesized monolayer bismuthene due to its instability. Chuang’s group was the first to predict the realization of 2D TIs via substrate modulation. The prediction had then been successfully synthesized and verified in 2017 [2]. The key to this brilliant design is the effect of substrate via chemical bonding. The planar bismuthene is a trivial insulator, but due to the bonding with the substrate, two extra electrons are provided to the system resulting in the shift of the Fermi level to a higher energy level where a band inversion is present due to strong spin-orbit coupling (SOC). This finding is considered a crucial breakthrough in 2D topological materials design.

**Fig. 1:**
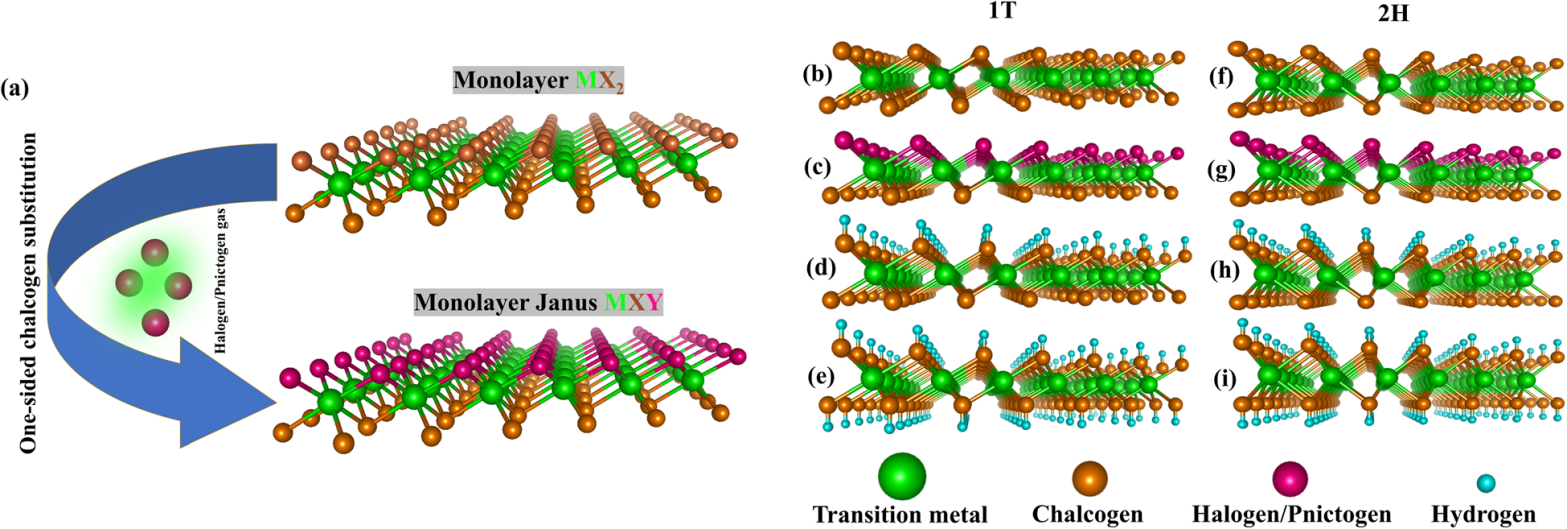
**(a)** Schematic diagram of the fabrication process of monolayer Janus MXY (X,Y = Halogen or Pnictogen) from the parent MX_2_ material. Structures of the 1T and 2H of (**b**, **f)** parent MX_2_ material, (**c**, **g)** Janus MXY material after halogen/pnictogen substitution. (**d**, **h)** One-sided hydrogenation, and (**e**, **i)** two-sided hydrogenation

Further, transition metal dichalcogenides (TMDs), with a chemical formula of MX_2_, have been stimulating a lot of research interest in the field of 2D materials because of their fascinating tunable properties that arise upon dimensional transition from bulk to the 2D regime. This raises the question whether the quantum spin Hall effect exists in these materials. Chuang’s group again had designed and demonstrated the tailoring of 2D TIs via functionalization and substitutional doping of monolayer TMDs. Figure 1 illustrates the structural engineering of 2D TMDs through halogen/pnictogen substitution, resulting in Janus 2D TMDs [3], and hydrogenation [4]. These methods significantly alter and manipulate their electrical, topological, and magnetic properties. The proposed Janus 2D TMDs, named after a Roman god with 2 faces, leads to symmetry breaking which results in novel characteristics and phenomena.

**Fig. 2:**
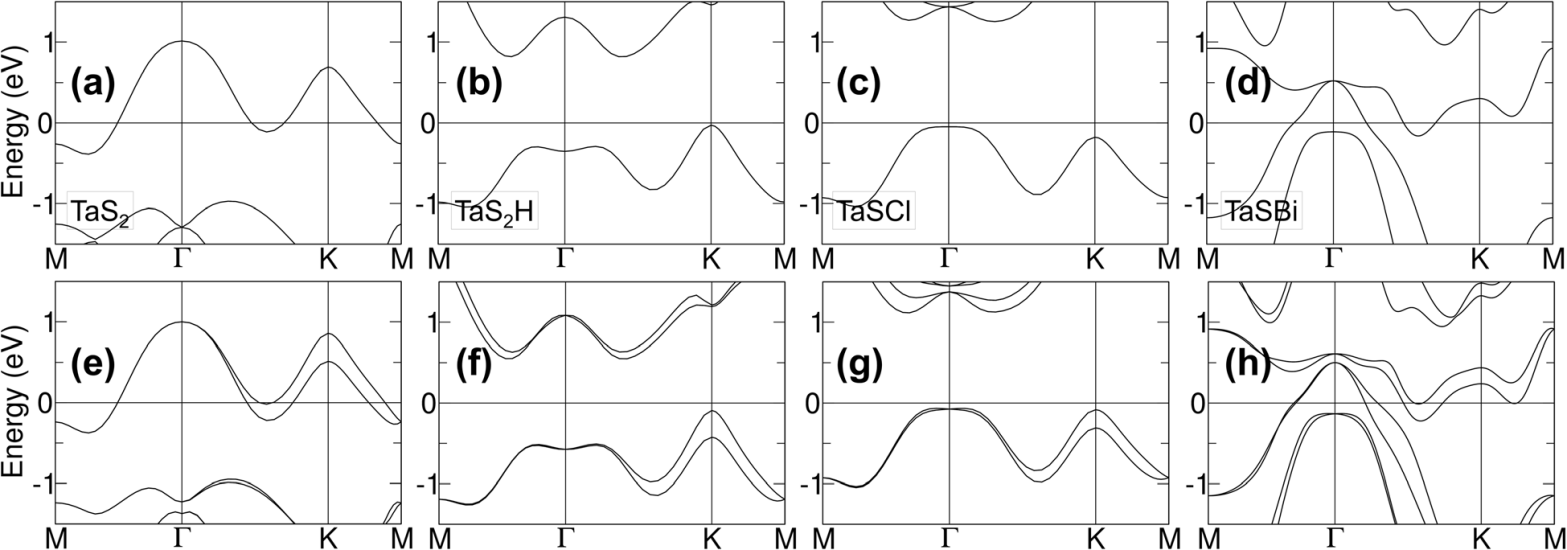
Band structures of the stable structural phases without SOC (**a**–**d**) and with SOC (**e**–**h**). (**a**, **e**) Monolayer pristine TaS_2_ film in the 2H structure. (**b**, **f)** One adsorbed hydrogen on TaS_2_ in the 2H structure. (**c**, **g**) One CI atom substituting an S atom in the 2H structure. (**d**, **h**) One Bi atom substituting an S atom in the 1T structure. Reprinted from Reference 3

Chuang’s group probed the topological properties of all the possible combinations of Janus 2D TMDs via halogen and pnictogen substitution, including one-sided hydrogen adsorption of MX_2_ (M = V, Nb, Ta, Tc, or Re; X = S, Se, or Te) films in both 1T and 2H structures [3] (see Fig. 1b–i). Using a high throughput approach, a total of 294 compounds were examined. Referring to Fig. 2a, e, TaS_2_ has an odd number of electrons, hence, the Fermi level crosses the highest half-occupied band. By substituting one chalcogen with one halogen, one extra electron is introduced, which could also be done by adsorbing one hydrogen on TaS_2_. Both induced an upward shift of the Fermi level resulting in a trivial phase as shown in Fig. 2b, c, f, g. Contrary to halogen substitution and one-sided hydrogenation, pnictogen substitution results in the loss of one electron, causing the downshift of the Fermi level. In the case of TaSBi film (without SOC), as seen from Fig. 2d, the VBM and CBM levels now touch at Γ. When strong SOC is included, a 108 meV gap opens at the Γ point as shown in Fig. 2h.

**Fig. 3:**
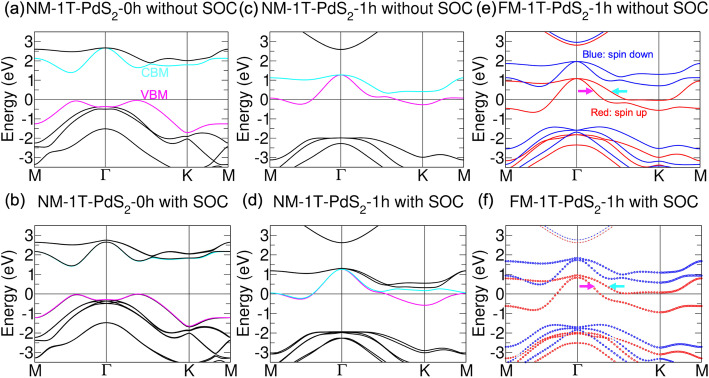
Calculated band structure under HSE06 of 1T PdS_2_-1h. (**a**, **b**) NM pristine 1T PdS_2_ without and with SOC (**c**, **d**) NM 1T PdS_2_-1h without and with SOC, and (**e**, **f**) FM 1T PdS_2_-1h without and with SOC. Reprinted from Reference 4

Moreover, the high throughput method was again used by Chuang’s group to study the electronic and magnetic properties of pristine and hydrogenated 1T, 1T’, and 2H TMD monolayers [4]. Group IV, VI, and X pristine TMD monolayers were found to mostly adopt 1T and 2H as their stable structures, except for WTe_2_ which exhibits 1T’, and has been identified as a topological insulator. Upon hydrogenation, a structural phase transition was also observed. Surprisingly, nineteen 2D magnetic materials were found through hydrogenation (see Fig. 1d, e, h, i). After the pre-screening of the materials using standard PBE-based calculations, further analysis of band topologies under hybrid functional calculations revealed that four of these identified magnetic monolayer structures exhibit quantum anomalous Hall effect (see Fig. 3). Here, PdS_2_ with one hydrogen passivation elevates the Fermi level where a band inversion is observed. Furthermore, this results in a strong ferromagnetic state with a non-trivial topological Chern number.

**Fig. 4:**
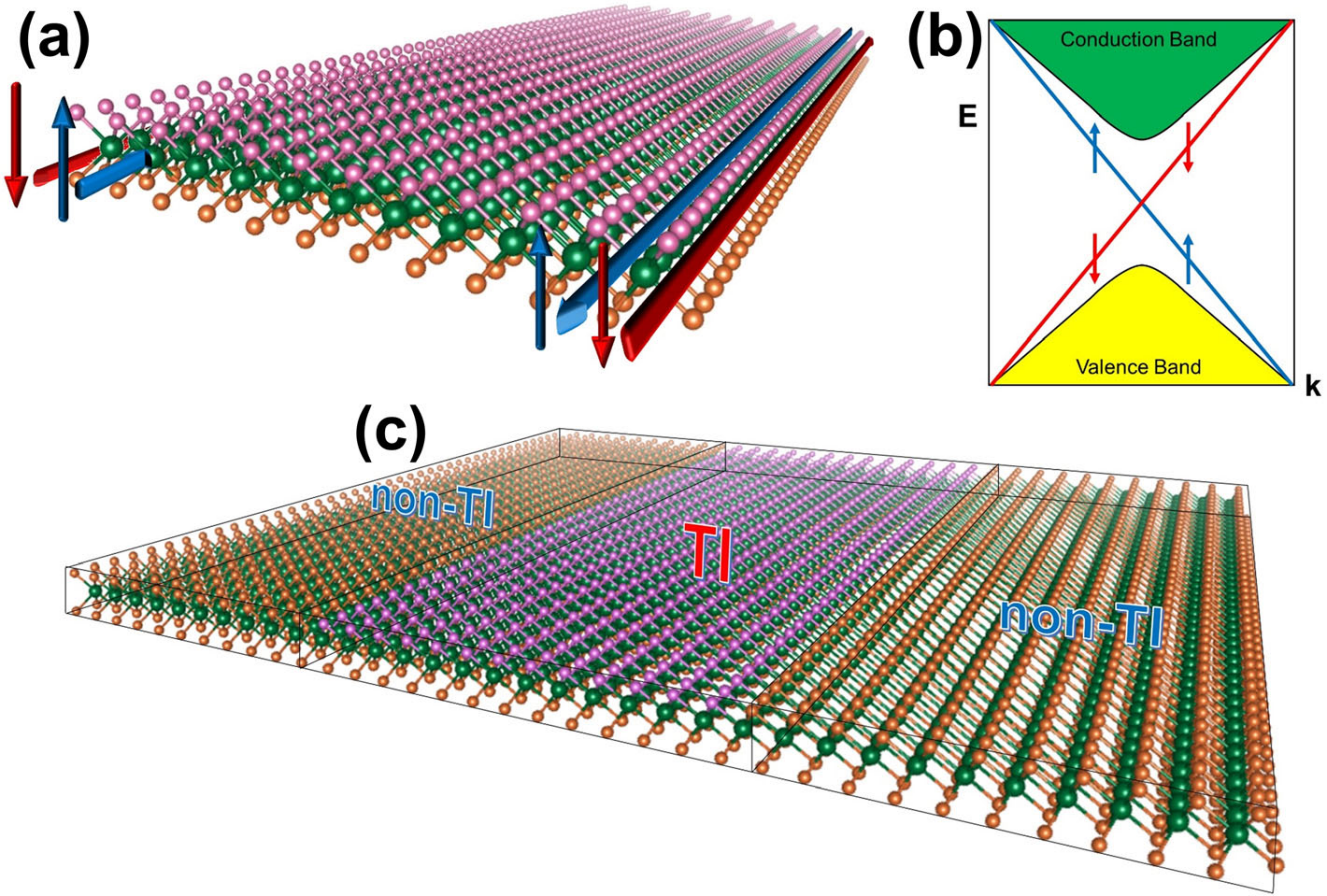
**(a**, **b)** Illustrations of the topologically protected edge states. (**c)** Illustration of possible synthesis of Janus 2D materials via lithography or etching method. Reprinted from Reference 3

To demonstrate the application of spin-transport in 2D TIs, Fig. 4a, b illustrates the topologically protected edge states in the real and reciprocal spaces of the quantum spin Hall (QSH) phases with the spin-up (blue) and spin-down (red) electron channels conducting along one edge of the ribbon**.** Figure 4c shows a schematic diagram of a sheet of TMD film with a region of 2D Janus to demonstrate the possibility of topological insulator phase upon substitutional doping. This design may be realized via lithography or etching method, a well-known method in the semiconductor industry used in fabricating integrated circuits and micro-electromechanical systems.

## Blending Superconductivity and Magnetism to Create Composite Quantum Excitations by Christos Panagopoulos and Alexander Petrović

**Fig. 5:**
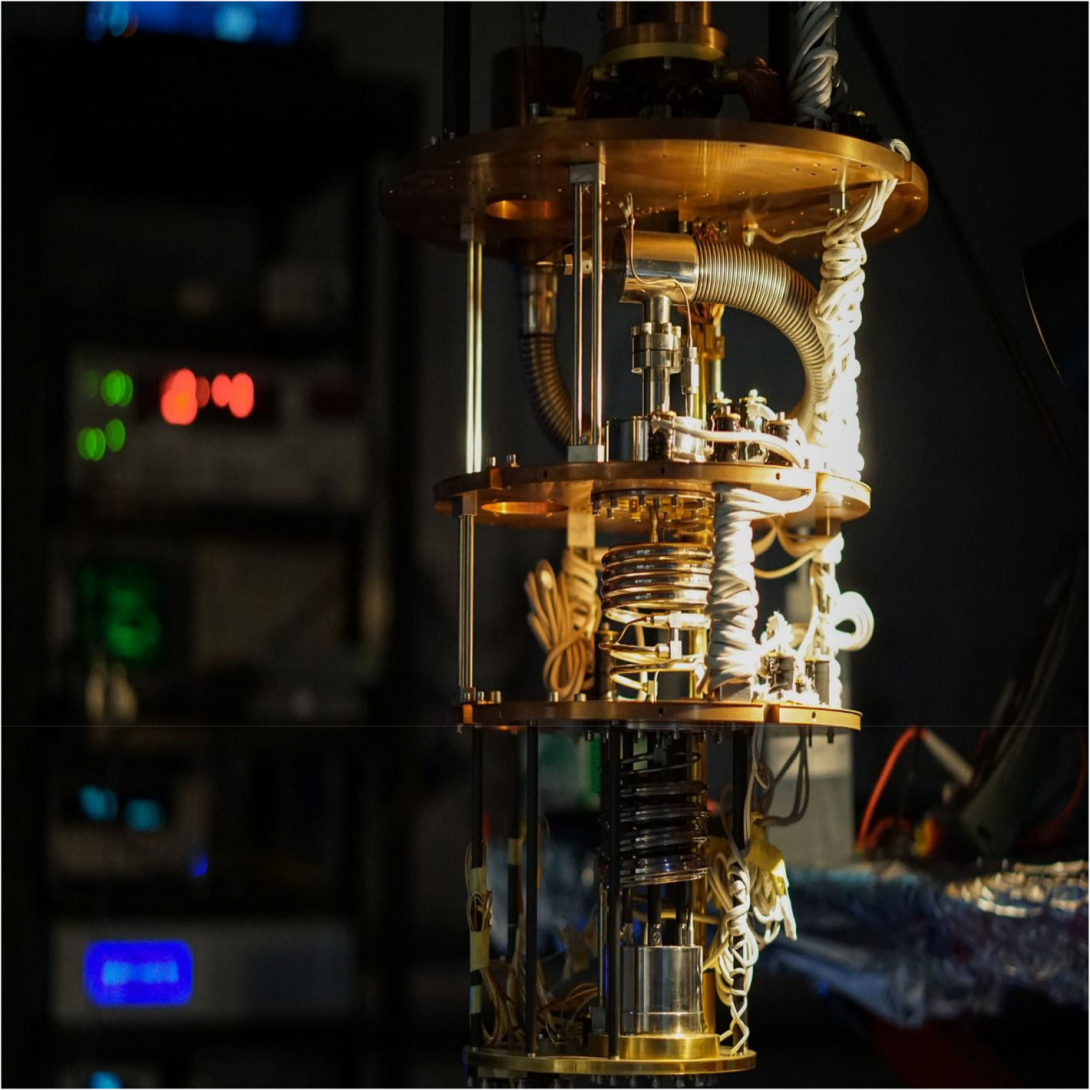
A novel material combining superconductivity with magnetism was developed and studied in Professor C. Panagopoulos’ laboratory. The cryostat pictured here maintains the material at milliKelvin temperatures while ultra-low noise electrical transport and spectroscopy measurements are performed

The team of Professor Christos Panagopoulos recently created a novel “hybrid” material in which superconductivity and magnetism interact via their respective topological solitons:vortices and skyrmions. This interaction is achieved by experimentally coupling chiral magnetism and superconductivity in [IrFeCoPt]/Nb heterostructures. They also detected a thermally tunable Rashba-Edelstein exchange coupling in the isolated skyrmion phase. This realization of a strongly interacting skyrmion-(anti)vortex system opens a new path toward controllable topological hybrid materials (Fig. 5).

Chiral magnets and superconductors host topological excitations known as skyrmions and vortices, whose duality was recognized in the 1980s [5]. Topological solitons are particle-like phenomena which are unusually stable against external perturbations. Just as a knot on a string cannot be undone by pulling on the ends of the string, a topological soliton cannot be easily destroyed by disturbing the material. While this may sound like an esoteric concept, various types of topological solitons are known to exist in different materials, from magnets to protein chains.

Two types of topological solitons which are of special interest to physicists are superconducting vortices and magnetic skyrmions. Vortices are topological solitons which appear in many superconductors—materials that conduct electricity with zero resistance—upon exposure to a magnetic field. They consist of tubes of magnetic flux, surrounded by nanometer-sized, tornado-like swirls of electric current. Skyrmions, on the other hand, are topological solitons which appear in certain magnetic materials. Ordinary magnets are composed of nanoscopic magnetic “spins” which all point in the same direction. In magnets displaying a chiral instability however, the spins prefer to twist relative to their neighbors rather than aligning, thus creating intricate patterns called skyrmions.

In a paper published in Physical Review Letters [6, 7], the team of Professor Panagopoulos announced the successful combination of two different topological solitons—vortices and skyrmions—in a material. This breakthrough is exciting because theoretical physicists have predicted that combining magnetism and superconductivity in this manner can yield Majorana fermions—exotic particles that act as their own antiparticles. If a superconductor is brought into proximity with a chiral magnet containing skyrmions, the field from the magnet is expected to change the symmertry of the electron pairing responsible for superconductivity, resulting in the formation of a “topological superconductor” whose vortices each contain a single Majorana fermion.

Majorana fermions have potentially revolutionary implications for quantum computing. In a Majorana-based quantum computer, information can be encoded using pairs of Majorana fermions (i.e., two vortices). Such pairs would be much more resilient to environmental disturbances than the quantum bits used in existing quantum computers.

A major challenge was that magnetic vortices and skyrmions do not simultaneously appear, let alone interact, in ordinary materials. To get around this, the team developed a hybrid material combining niobium, a known superconductor, with an atomically-precise magnetic multilayer made of iridium, iron, cobalt and platinum.

This is a completely new material architecture exploiting a stack of magnets and a superconductor. The carefully-designed properties of this structure allow coupling between topological solitons from two distinct quantum orders—chiral magnetism and superconductivity—for the very first time.

**Fig. 6:**
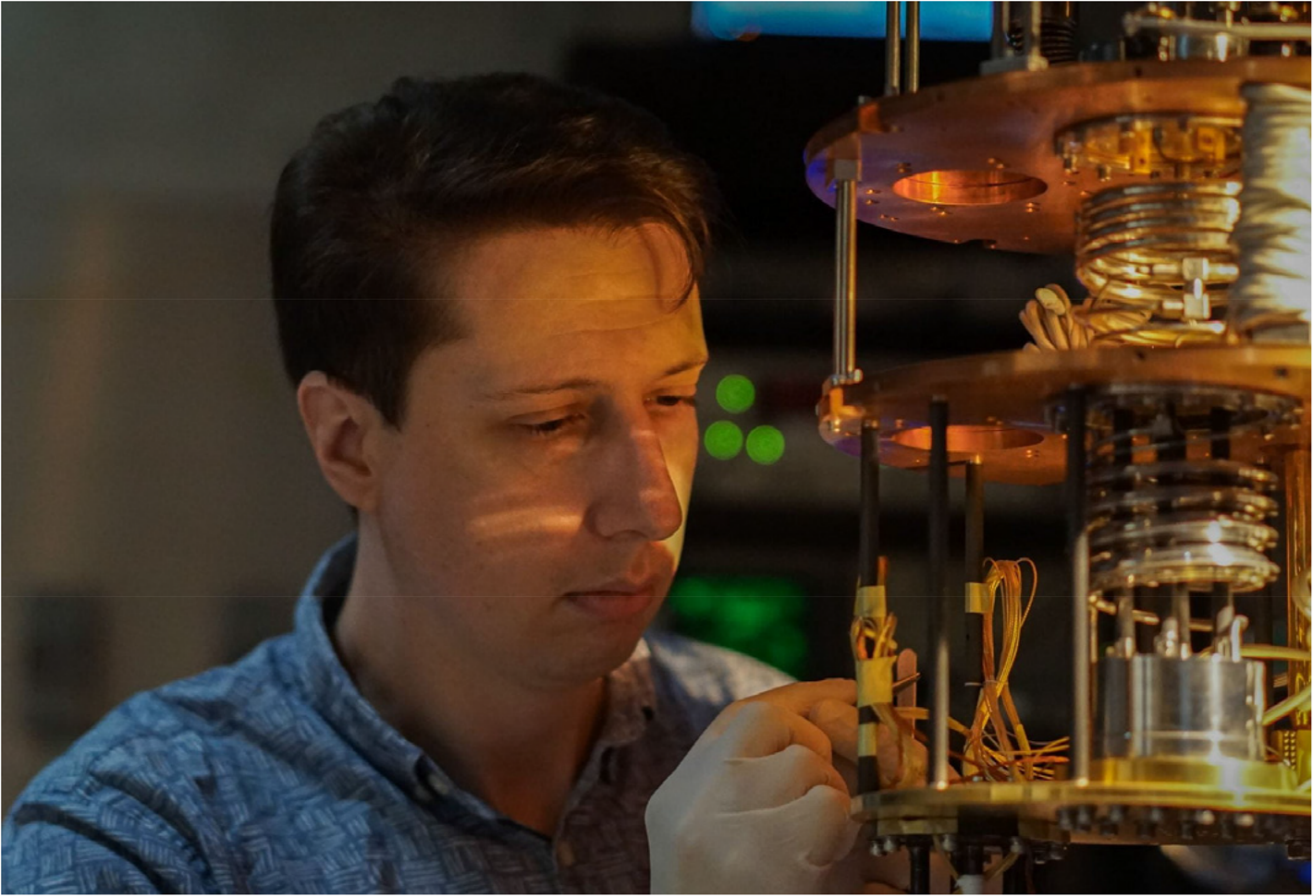
The first author of the article, Dr. Alexander Petrović at work on the experimental apparatus

The properties of the new material were investigated using many different scientific methods in a joint effort by researchers in Nanyang Technological University, Singapore, the University of Geneva in Switzerland, the Technion in Israel, and the University of Antwerp in Belgium. Each of the different analytical techniques was chosen to expose a different aspect of the hybrid behavior. Electronic snapshots of the superconducting vortices were taken using scanning tunneling spectroscopy at temperatures a fraction of a degree above absolute zero, while magnetic force microscopy tracked the formation and morphological evolution of skyrmions within the magnetic layer. An extremely sensitive detector known as a Superconducting Quantum Interference Device (SQUID) captured the nucleation of vortices by skyrmions, while the influence of skyrmions on the flux dynamics was deduced from the tiny voltages induced by vortices moving in an applied electrical current (Fig. 6).

Computer simulations of the material matched the key experimental results, hence verifying the ability of individual skyrmions to generate vortices in the neighboring superconductor. These simulations also exposed intriguing flow characteristics for the vortices at high currents, which could enlighten studies of particle dynamics in other complex systems.

The beauty of these [IrFeCoPt]/Nb heterostructures is that they show stable vortices above elongated chiral spin textures, as well as isolated skyrmions. This is an ideal geometry in which to attempt manipulation of individual vortices, a prerequisite for computational operations. In the future, the team aims to explore the electronic properties of their samples for signatures of the anticipated Majorana fermions.

## 17th Italian-Korean Symposium for Relativistic Astrophysics by Stefano Scopel

Due to the COVID-19 pandemic, this year the 17th Italian-Korean Symposium for Relativistic Astrophysics was held from August 2 to 6 in a hybrid form: offline at Kunsan University, and online on Zoom.

The Italian-Korean Symposium is one the longest-running programs between the Asian countries and Europe. It was initiated in 1987 in Hanyang University and has since been held every two years, with the site alternating between Italy and Korea. For its entire history, the Italian organizer of the meeting has been Remo Ruffini, from The University La Sapienza/Rome and ICRANet/Pescara. This year, the main Korean organizer was the Center for Quantum Space Time (CQUeST) institute of Sogang University. The Symposium was also sponsored by the International Center for Relativistic Astrophysics (ICRA), the ICRA network (ICRANet), and the Asian Pacific Center of Theoretical Physics (APCTP), and also received the endorsement of the Division of Astrophysics, Cosmology and Gravitation of the Association of Asia Pacific Physical Society (AAPPS).

The symposium opened with a welcoming address given by president of Kunsan National University Byeong-Sun Kwak, and this was followed by a congratulatory address from Federico Failla, the Ambassador of Italy in Korea, after which Remo Ruffini gave participants and guests an overview of the history of the meeting. Failla stressed in particular the symposium’s important role in establishing and strengthening scientific relations between Italy and Korea, while Ruffini expressed his hope to intensify relations between ICRANet and CQUeST and Sogang University. In his closing remarks on the last day of the meeting, director of CQUeST Stefano Scopel confirmed that the center will continue to support the symposium in its future editions and will be signing a cooperation protocol with ICRANet in the near future to facilitate exchanges of students and researchers.

The IOC was formed by Remo Ruffini (ICRA-Sapienza University Rome/ICRANet, Co-Chair), Rong-Gen Cai (ITP, China), Pisin Chen (LeCosPA, National Taiwan University), Misao Sasaki (IPMU, Japan), Jun Luo (Sun Yat-Sen University), Sang Pyo Kim (Kunsan National University), Bum-Hoon Lee (CQUeST, Sogang University, Chair), Changhwan Lee (Pusan National University) and Hyungwon Lee (Inje University). Members of the LOC were Wontae Kim (CQUeST, Sogang University), Jeong-Hyuck Park (CQUeST, Sogang University), Wonwoo Lee (CQUeST, Sogang University), Jin Young Kim (Kunsan National University), Jiwan Kim (Kunsan National University), Bogeun Gwak (Dongguk University), and Imtak Jeon (APCTP).

The meeting was attended by approximately 50 physicists, including offline and online participants, with a program of 28 plenary talks on Astrophysics, Cosmology, and Gravitation. Speakers included Remo Ruffini (Rome University La Sapienza/ICRANet), Dong-Hoon Kim (Seoul National University, Korea), Lang Liu (Institute of Theoretical Physics, China), Chen-Te Ma (APCTP, Korea), Daniele Gregoris (Jiangsu University of Science and Technology, China), Soroush Shakeri (Isfahan University of Technology, Iran), She-Sheng Xue (Rome University La Sapienza/ICRANet), Mu-In Park (Sogang University, Korea), Lu Yin (Sogang University, Korea), Chan Park (National Institute of Mathematical Science, Korea), Chang-Hwan Lee (Pusan National University, Korea), Eoin O Colgain (Sogang University, Korea), Rahim Moradi (ICRANet, Italy), Carlos Raul Arguelles (ICRANet, Italy), Wonwoo Lee (CQUeST, Sogang University), Dong-han Yeom (Pusan National University, Korea), Jin Young Kim (Kunsan National University, Korea), Pisin Chen (National Taiwan University and Stanford University, USA), Maria Giovanna Daniotti (ICRANet, Italy), Liang Li (ICRANet, Italy), Hochoel Lee (Sogang University, Korea), Myeonghwan Oh (Kyungpook National University, Korea), Davood Rafiel Karkevandi (Isfahan University of Technology, Iran), Narek Sahakyan (ICRANet, Italy), Jorge Rueda (ICRANet, Italy), Sung-Won Kim (Ewha Womans University, Italy), Mahdis Ghodrati (APCTP, Korea), and Sang Pyo Kim (Kunsan National University, Korea). Presentations will be published by the proceedings of the Journal of the Korean Physical Society (JKPS).

The Italian participants and all other participants who were not based in Korea joined the meeting via Zoom conference calls. Due to the time difference between Italy and Korea, the meeting was held every afternoon starting from 4pm. In parallel with the Italian-Korean Symposium, CQUeST organized the Workshop for Cosmology and Quantum Space Time 2021 (CQUeST 2021) that was held every morning during the same week.

As the two meetings had similar target audiences, joint participation was encouraged by distributing the same Zoom link for both.

**Fig. 7:**
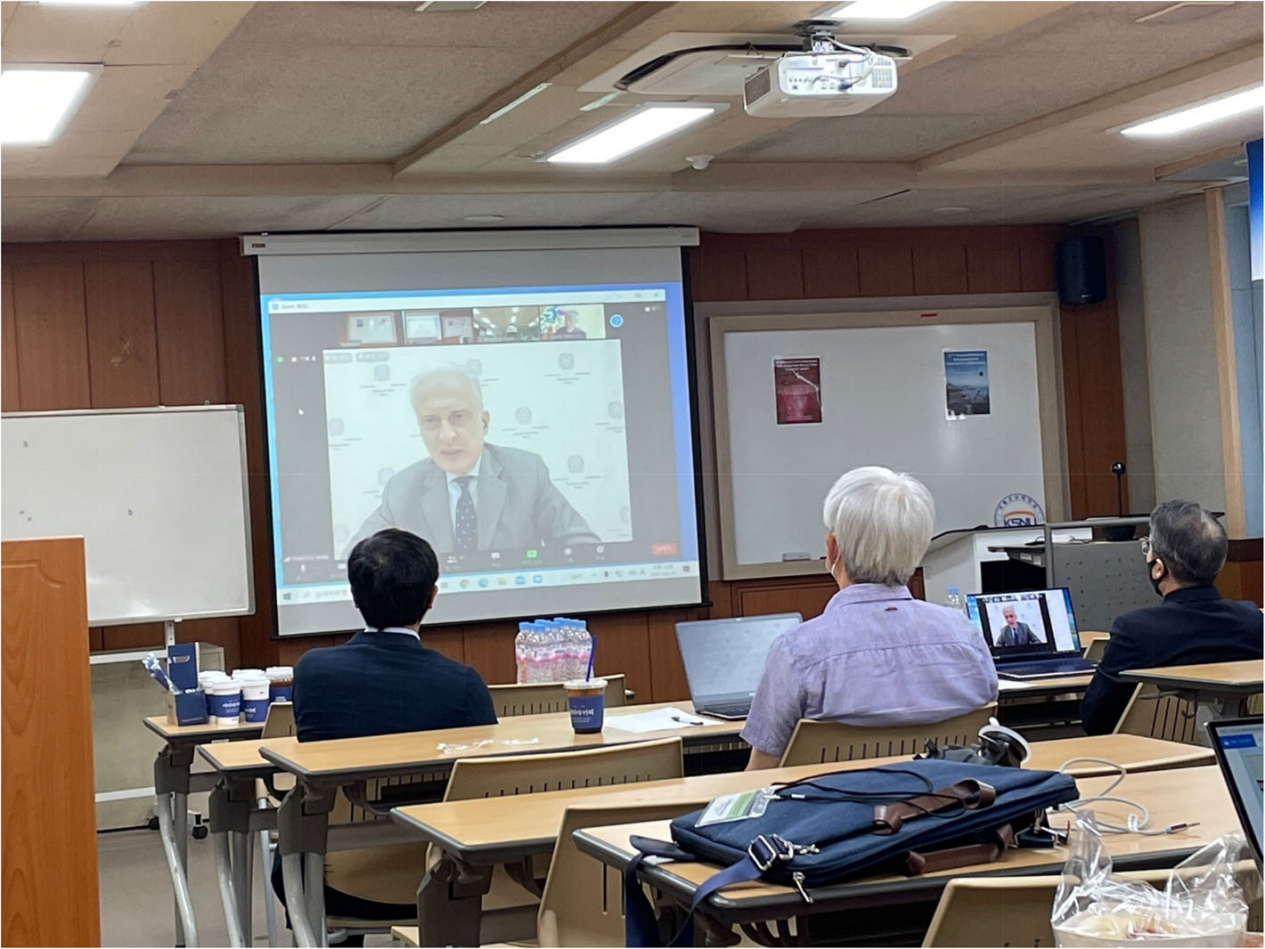
H.E. the Ambassador of Italy Federico Failla addresses the opening session of the symposium via video conference. Professor Byeong-Sun Kwak, the President of Kunsan University, is sitting at the far right of the picture

**Fig. 8:**
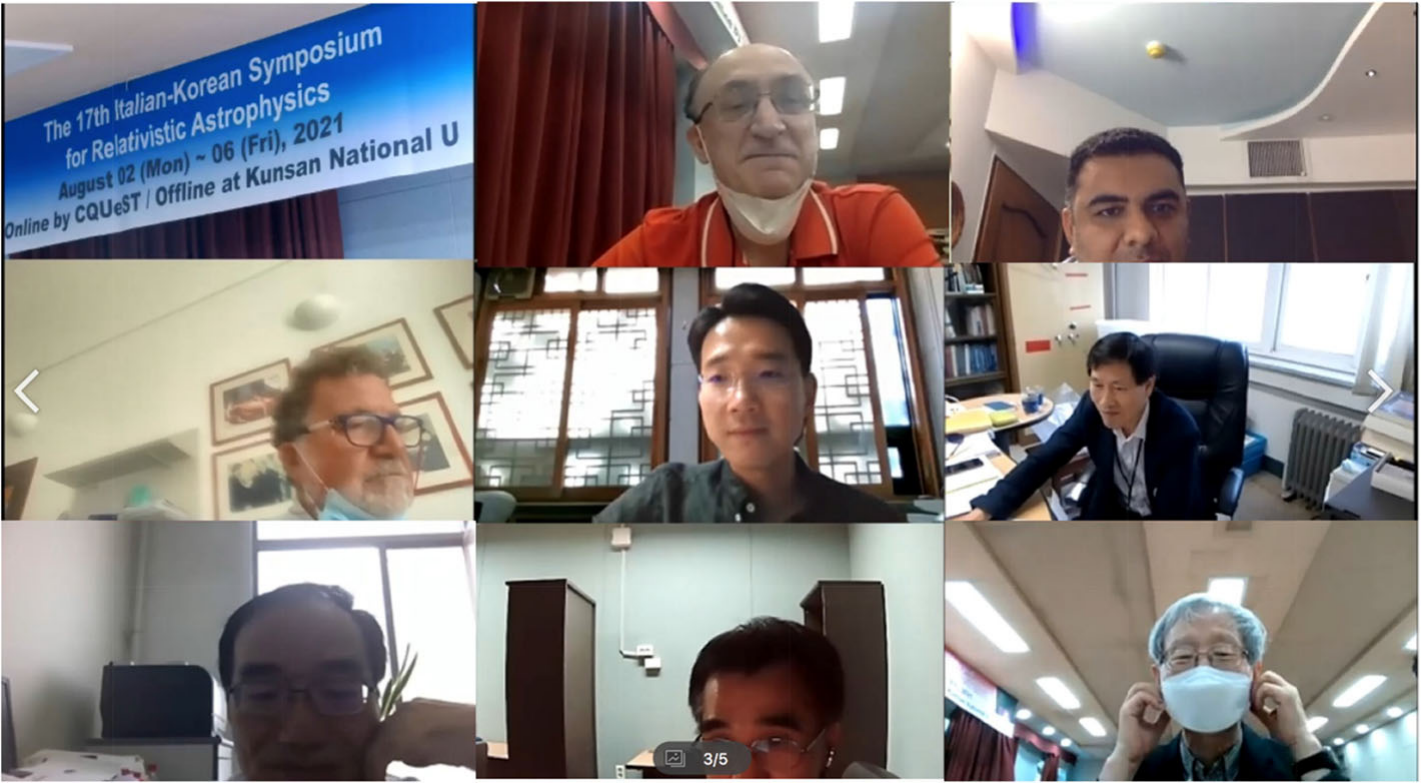
Screen capture from the Zoom session of the meeting

The next Italian-Korean Symposium for Relativistic Astrophysics will be held in Italy in the summer of 2023 (Figs. 7 and 8).

## Report on the 45th AAPPS Video Council Meeting by AAPPS

The 45th Council Meeting of the Association of Asia Pacific Physical Societies (AAPPS) was held online from 2:00 p.m. to 4:03 p.m. KST (Korea Standard Time) on March 3, 2021, via Zoom session hosted by the Asia Pacific Center for Theoretical Physics (APCTP). The participants were Jun'ichi Yokoyama (president), Hyoung Joon Choi (vice president), Nobuko Naka (secretary), Keun-Young Kim (treasurer), Gui-Lu Long (ex officio member as a former president), and council members Tao Xiang (the Chinese Physical Society (CPS)), Mio Murao (the Physical Society of Japan (JPS)), Kurunathan Ratnavelu (Malaysian Institute of Physics), Fu-Jen Kao (the Physical Society located in Taipei), and Meng-Fan Luo (the Physical Society located in Taipei).

The meeting was observed by Eunjeong Lee (AAPPS liaison officer). Council members Jodie Bradby (Australian Institute of Physics (AIP)), Xiu-dong Sun (CPS), Akira Yamada (the Japan Society of Applied Physics (JSAP)), Rajadeep Singh Rawat (Institute of Physics Singapore), Ruiqin Zhang (the Physical Society of Hong Kong), Woo-Sung Jung (KPS, APCTP), and Nguyen Quang Liem (Vietnam Physical Society) were absent.

1. Secretary Naka reported the presence of 10 council members out of 17 members, and the quorum was not fulfilled. It was confirmed that the minutes of this council meeting should be circulated by email for approval.
2. President Yokoyama opened the 45th Council Meeting and welcomed the participants. The agenda was adopted as prepared by the president.
3. Vice President Choi explained the proposal of providing one-time support to the Division of Condensed Matter Physics (DCMP). He stated that this one-time support would be separate from the annual support to DCMP and could be used for preparing their website, their first division meeting, and whatever else they might need, without submission of receipts. Typically, when a new division has been established, the AAPPS Council has provided one-time support to the division. Previously, support of $2,000 USD was provided to the Division of Plasma Physics (DPP) in 2015, to the Division of Astrophysics, Cosmology, and Gravitation (DACG) in 2016, and to the Division of Nuclear Physics (DNP) in 2017. In 2015 and 2016, the financial support for the two divisions was allocated from donations by APCTP, which had no specific limits for its use. Vice President Choi was unsure about the source of the one-time support to DNP in 2017. Considering that the foundation of another new division, i.e., the Division of Particles and Fields (DPF), is underway, the coordination of one-time support to DPF also will become necessary soon. The AAPPS membership fees that have not been used in the past 2 or 3 years could be used to provide one-time support to DCMP.

Choi asked about the financial balance of AAPPS this year, and Lee stated that it is $52,820 USD. Choi confirmed that we have a carryover of $50,000–$60,000 USD this year, in addition to the donation from the Leo Koguan Foundation. Yokoyama stated that the financial aspects of providing one-time support do not cause any problems for the sustainable operation of the association and the present proposal by Choi is reasonable. As for the annual support to division activities from APCTP amounting to 30M KRW, the AAPPS Council already decided to financially support DCMP more, in the first year, than the three other already existent divisions.

The proposal was approved by the present council members. Yokoyama asked Choi to discuss with the DCMP chair how to transfer the money, if the absent members support the proposal.

(4) Kao, the chair of the selection committee of the CN Yang Award 2020 reported on the preparations for the CN Yang Award 2021. First, the timeline for the selection process was introduced. The call for nominations was made on February 26th last year, so the next announcement will be sent to individual societies and APPC14 participants soon after being approved in this council meeting. Second, Kao explained the two-step selection process. APCTP sends the nominees’ information to the AAPPS divisions for prescreening. Nominations unrelated to AAPPS divisions are reviewed by the sub-selection committee. By June 18th last year, the preselection results of the sub-selection committee were submitted to the selection committee. The selection committee finalizes three awardees. In 2021, we will have maximally 12 recommendations from four divisions and several recommendations from the sub-selection committee at the final phase after prescreening. Third, Kao proposed amendments to the rules. The major proposed changes are as follows: (i) AAPPS divisions can nominate up to three candidates. We would have an option to invite selection committee members external to the AAPPS Council if they were approved by the council. [*Note: We wish to continue the discussion on amendment (i), and the new nomination channel is supposed to be open starting in 2022.] (ii) For reference information, a provision of citation numbers is preferred. This is intended to be used for the relative positioning of a nominee within the same specific field and not for comparison among nominees in different categories. (iii) The maximum number of nominations after prescreening by each AAPPS division has been increased from one to three. (iv) The maximum number of selection committee members has been increased from 9 to 12. A division sending a member to the selection committee should have at least one nominee.

Kao added that we have already discussed these issues in the council meeting held last December, and that we need to shift the rules to simply reflect the changes according to the increasing number of divisions. Xiang mentioned that the physical societies of each country/region and APPC participants can also make nominations. Yokoyama added that we should be proud that the selection process for the CN Yang Award is highly sophisticated and accurate. Kao added that experts of each division carefully examine how much contribution is made by a candidate if the candidate is one of many authors of a group work. To confirm the amendments of the rules and the timeline for this year, Xiang proposed to ask for approval of all the council members, not just the participants of this online meeting. Yokoyama summarized that approval was obtained from the present council members. He proposed that Kao remains as the selection committee chair for one more year until his 3-year term is fulfilled; he has already served as the chair for the past 2 years. This proposal was also approved by the present council members. Kao responded that he would try his best to have the selection process be completed in a timely and orderly manner.

(5) Choi stated that the start of scientific sessions at APPC15 is scheduled for 22 August 2022 in Gyeongju, Korea. The number of rooms for the conference will be similar to that of APPC14. Regarding the timeline, the first announcement will be made by June 30th, 2021, which is 14 months before the conference.

Choi started a discussion on how to form the committees for APPC15. Long recommended Xiang as one of the two deputy chairs of the organizing committee and the co-chair of the program committee. Xiang accepted this recommendation. Yokoyama mentioned that most of the past APPCs have been held in the country/region where the AAPPS president was located. This was not the case for APPC14 and APPC15. Prof. Long, the president at that time, was the chair of the advisory committee for APPC14. At the APPC held in Australia, the AIP president served as the chair of the organizing committee. Choi proposed for the advisory committee to be chaired by the AAPPS president, and the organizing committee to be co-chaired by the KPS president and AAPPS president, and that he would volunteer as the chair of the program committee. Yokoyama mentioned that he can sit on only one chair at one time and proposed that Choi take a more prominent position, namely, the chair of the organizing committee. It was agreed that we would wait for the internal discussions in KPS to take place before discussing the matter further.

Yokoyama also suggested for Yamada from JSAP and a member from the Physical Society located in Taipei to be co-chairs of the program committee, whose expertise is not covered by the AAPPS divisions. Jung was recommended to become the chair of the local organizing committee as Pohang University of Science and Technology (POSTECH) is close to the conference venue. To summarize, the following members were temporally suggested.

*Advisory Committee Chairs: Current AAPPS president, APCTP president

*Organizing Committee Chair: KPS president

*Organizing Committee Deputy Chairs: Hyoung Joon Choi, Tao Xiang

*Program Committee Chair: Hyoung Joon Choi

*Program Committee Co-chairs: Tao Xiang, Akira Yamada, Fu-Jen Kao

*Local Organizing Committee Chair: Woo-Sung Jung

*Local Organizing Committee Members: Hyoung Joon Choi, Keun-Young Kim, Korean researchers

Choi explained that this event will be held by AAPPS and KPS. He will discuss with the KPS president to know if KPS would directly commit. Afterwards, he will discuss with President Yokoyama and circulate the list of members by email.

Yokoyama asked how many plenary talks are planned. Choi answered that we will follow the APPC14 schedule but will not invite a Nobel laureate. We need to discuss and finalize the list of plenary speakers in 2 or 3 months. The list will be distributed by email. Yokoyama mentioned that he hopes the four divisions will play leading roles by programming talks in their respective areas of expertise for this event. Choi stated that we assume that COVID-19 will end this winter or at least next spring, before APPC15. Changing the schedule is not a good idea and switching the events to an online format should be considered, in case the need should arise. The deadline to decide whether the conference is going to be held in person or online would probably be 3 months prior to APPC15, as dictated by the cancellation rules for booking the hotel and convention center. Choi will confirm the deposit as any financial loss should be avoided. Concerning the financial aspect of the conference, no support will be provided for invited speakers. Ratnavelu stated that at APPS14, the registration fee was $500 USD and two meals were included in the package of $40 USD per day for one participant.

Choi summarized that we will proceed with the preparations for APPC15, in close discussions with KPS.

(6) Long, the editor-in-chief of the AAPPS Bulletin (*AB*), reported on the status of *AB* and his thoughts. As a brief history, he introduced past five editor-in-chiefs, the first issue published in June 1991, and a memorandum regarding *AB* between KPS, APCTP, and AAPPS. There were some changes during the term (2017–middle 2020) of the former editor-in-chief, Prof. Motobayashi, who added a new section called “Review and Research”. This year, cooperation with Springer Nature began and five articles were already published.

Long emphasized that journals are the banners for associations. AAPPS performs poorly in this aspect compared to its counterparts in America and Europe, although AAPPS supports APPC, the CN Yang Awards, and division activities. In Google Scholar Citations, only 27 papers were cited among 132 papers published in *AB*. The ideal new format of *AB* is a comprehensive journal, similar to *Nature*, *Science*, and *Science Bulletin.* The number of publications is supposed to be 30 or less than 100 per year, which is a small portion compared to the thousands of articles published in a specialized journal or a member society journal.

Long explained that cooperation with Springer Nature was one of the three options. The editorial board was reorganized and the new sections, “News and Views” and “Research and Review”, were launched. The *AB* ranking, intended to compensate for the discontinued section “Institutes in Asia Pacific”, was decided to be cancelled after consultation with senior editors. The tasks of *AB* are to increase the quality of papers, to publish more research highlights, to get indexed in databases, to increase its impact factor, and to increase the number of issues (from 6 to 12 in 6–7 years) as a world-leading physics journal. Long said that in order to achieve these goals we need to act now, and asked the presenting council members to agree with the position of *AB*.

Ratnavelu asked if the cooperation with Springer Nature was proposed or decided. Long answered that the contract was already made. Yokoyama added that the agreement was inappropriately signed, so that it could not work as an official document of AAPPS. According to the report, the article processing charge (APC) should be paid and AAPPS cannot sustain this new style in cooperation with the publishing company. The contract is for 5 years, while the Korean government promised to provide support only for 1 year, namely on an annual basis. The government will continue to provide support as long as the journal is performing well. APCTP was trying to obtain extra funding to launch the new style, and the APCTP president is correcting the situation. Long added that if APCTP cannot support the APC anymore, it could be managed by the *AAPPS Bulletin*’s independent account for 2 years. Yokoyama informed that a meeting is going to be held to explain the situation to five member societies supporting the *AB*. Long voiced that the journal itself should be a leading journal. Editors from member societies are trying hard to collect papers, and in particular, the nuclear physics and the accelerator field have done an excellent job. These new articles are published in the previous style as well, and are citable with the volume number plus single-digit article number or the digital object identifier (DOI). There are now two websites for *AB*; the new one is managed by Springer Nature while the old one maintained by APCTP will also continue.

Kao commented on the advantages of cooperation with Elsevier. In the case of an editorial in Taipei, journal citations dramatically increased after cooperation with Elsevier. Many citations started to come from bundled journals and a network of heavy citations is promoted with the increased number of submissions. The contract with Elsevier was free for the first 6 years. The journal is already in Q2 category and making a profit which could be shared. K.Y. Kim was curious about promotion and asked whether Elsevier has a good strategy. Kao stated that the promotion impact can increase very quickly with the right combination of a journal and a publisher. Long mentioned that Springer Nature also has promotion measures and the articles are freely accessible; thus, he believes a large change will occur in the 3 years to come.

(7) President Yokoyama announced that a forthcoming council meeting is planned to be held in Hong Kong in December. He noted that we should carefully examine the possibility of having the meeting in person. He proposed to have the next online council meeting in summer and closed the meeting.

## Report on the 46th AAPPS Video Council Meeting by AAPPS

The 46th Council Meeting of the Association of Asia Pacific Physical Societies (AAPPS) was held online from 5:00 p.m. to 7:27 p.m. KST (Korea Standard Time) on 12 July 2021, via a Zoom session hosted by the Asia Pacific Center for Theoretical Physics (APCTP). The participants were Jun'ichi Yokoyama (president), Hyoung Joon Choi (vice president), Nobuko Naka (secretary), Keun-Young Kim (treasurer), Gui-Lu Long (ex officio member as a former president), and council members Tao Xiang (the Chinese Physical Society (CPS)), Xiu-dong Sun (CPS), Ruiqin Zhang (the Physical Society of Hong Kong), Mio Murao (the Physical Society of Japan (JPS)), Akira Yamada (the Japan Society of Applied Physics (JSAP)), Kurunathan Ratnavelu (Malaysian Institute of Physics), Rajadeep Singh Rawat (Institute of Physics Singapore), Fu-Jen Kao (the Physical Society located in Taipei), Meng-Fan Luo (the Physical Society located in Taipei), and Nguyen Quang Liem (Vietnam Physical Society). Present as observers were Takhee Lee as a representative of the Korean Physical Society (KPS) for APPC15, Sang Pyo Kim (chair of the Division of Astrophysics, Cosmology, and Gravitation (DACG)), Bogeun Gwak (secretary of DACG), Je-Geun Park (chair of the Division of Condensed Matter Physics (DCMP)), S.M. Yusuf (vice chair of DCMP), Hiroyuki Nojiri (vice chair of DCMP), Weiping Liu (chair of the Division of Nuclear Physics (DNP)), Youngah Park (chair of the Women-in-Physics Working Group of AAPPS), Eunjeong Lee (AAPPS liaison officer), and Chae Young Lee (AAPPS editorial staff). Council members Jodie Bradby (Australian Institute of Physics (AIP)) and Woo-Sung Jung (KPS, APCTP) were absent.

(1) Secretary Naka reported the presence of 15 out of 17 council members, and the quorum was declared as fulfilled.

(2) President Yokoyama opened the 46th Council Meeting and welcomed the participants, including the division chairs and the KPS president. The participants introduced themselves in alphabetical order, based on the name of the country/region they were representing.

(3) The agenda was adopted as prepared by the president.

(4) Summary of previous meeting and email communications

President Yokoyama summarized the 45th Council Meeting held in March. While the meeting did not fulfill quorum requirements, affirmative replies were obtained from all council members by email.

In the meeting, the provision of one-time support to DCMP was approved following the proposal made by Vice President Choi. The money ($2,000 USD for the foundation of the new division) has already been sent to DCMP. Division Chair J.-G. Park expressed his gratitude for the support. Due to the increased number of divisions, amendments to the selection rules for the CN Yang Award were proposed and approved. In detail, the maximum number of selection committee members was increased from nine to 12. The role of divisions is to prescreen candidates, while nominations are made through member societies or participants in the most recent APPC, who are regarded as virtual individual members of the AAPPS. The question remains as to whether nominations via other channels would be accepted or not. APPC15 will take place in August 2022, and we have selected the advisory, organizing, program, and local organizing committee chairs and co-chairs. Yokoyama has asked all council members to serve on whichever committees they prefer. At the beginning of this year, cooperation with Springer Nature for the publication of AAPPS Bulletin (AB) was initiated. They have been publishing “Research and Review” including original research articles and “News and Views.” They discontinued the “Institutes in Asia Pacific” section. Of the five member societies that were contributing to AB, JSAP decided to stop their support for AB and instead began providing annual support to the main body of AAPPS. APCTP will take responsibility for covering the article processing charge (APC) for invited review articles. A new draft of the amendment to the contract arrived and was circulated to the council members by email.

(5) Preparations for APPC15

Vice President Choi explained that APPC15 will be a 5-day conference starting on 22 August 2022 in Gyeongju, Korea. Currently, 14 meeting rooms, one exhibition hall, and one convention hall have been booked. Regarding the timeline, an ordinary general meeting and council meetings are planned for 21 August. The first announcement was originally planned to be sent by 30 June 2021, but this deadline is to be extended to the end of July 2021. Recommendations by societies and divisions are needed for the program. The members of each committee were suggested as listed below.

*Advisory Committee

Chairs: Current AAPPS president and APCTP president

Members: Former AAPPS presidents, Presidents of member societies

*Organizing Committee

Chair: KPS president

Deputy Chairs: Hyoung Joon Choi and Tao Xiang

Members: Current AAPPS council members, Division chairs

*Program Committee

Chair: Hyoung Joon Choi

Co-chairs: Tao Xiang, Akira Yamada, and Fu-Jen Kao

Members: Recommended by societies and by division chairs

*Local Organizing Committee

Chair: Woo-Sung Jung

Members: Hyoung Joon Choi, Keun-Young Kim, and Korean researchers

Gui-Lu Long expressed that division secretaries should help division chairs in preparing the program. He also suggested changing the names of the organizing and advisory committees to the international organizing and international advisory committees, respectively. S.P. Kim commented that previously, the president at the time of the AAPPS foundation (C.N. Yang) and former AAPPS presidents were on the international advisory committee. Choi will reconsider committee members after checking those for APPC14 and APPC12. Further communications will take place over email.

Nguyen Quang Liem wondered if the international advisory committee would consist of members of AAPPS only. Choi answered that we might invite some well-known physicists or renowned figures as committee members, based on discussion after any recommendation. Y. Park reminded Choi that there had been an agreement to ensure sufficient representation of female physicists. Choi answered that we would be careful to consider gender equality when deciding the final members of committees.

Choi explained that the timetable of APPC15 basically follows that of APPC14, but includes one more day (5 days in total). He is considering having in the range of 15 to 20 plenary talks, depending on the number of participants. If we have plenty of invited and contributed talks, we may reduce the plenary slots to 15, or half plenary sessions may be considered.

Choi has started efforts to find a company to handle the conference and its website. The website will receive abstracts and registrations. It will also be used for producing certificates and for the program committee to access the abstracts to determine acceptance or rejection of the papers.

Akira Yamada asked about the expected number of participants and papers. Choi already discussed the issue with KPS, and at the previous Council Meeting. His first goal is 1000 participants, which would allow the conference to break even. The conference center is large enough to accommodate more participants, up to 1400. Hiroyuki Nojiri reported that the 29th International Conference on Low Temperature Physics will be held in Sapporo, Japan, from 18 to 24 August 2022, as one of the International Union of Pure and Applied Physics (IUPAP) supported conferences.

J.-G. Park wondered how much autonomy each division will be given regarding programs. He asked whether a division should act as a part of the program committee or if it would be under the control of the main programs of APPC. Choi answered that the only constraints are the starting and ending times. When a parallel session is assigned to a division, it will be fully organized by the division. He suggested forming program committees inside respective divisions and listing the members on the main homepage of APPC15, although such a record of those who contributed to the programming in each division for APPC14 had been lost. J.-G. Park asked if a poster session would be allowed when parallel sessions are assigned to each division to organize. Choi responded that selection between oral and poster presentations is also a division’s choice. We will have a list of topics assigned to respective divisions.

S.M. Yusuf expressed his concern. If the conference will be held online, we need to think about how to improve the poster sessions, which seem to have not provided good opportunities for discussion and feedback. Choi hopes that APPC15 will be held in a face-to-face manner. If COVID-19 still appears to be a problem, however, they will switch to a hybrid or fully online format.

Yokoyama asked how the nomination of plenary speakers from divisions is progressing. We will ask the member societies, council members, and divisions to nominate plenary speakers. Depending on the number of participants, some number of plenary speakers will be assigned to each division. Choi asked for opinions on reducing the number of plenary talks from 20 to 15. He explained that 2 h each day for plenary talks might be too long. As we assigned 16 h at the 4-day conference of APPC14, he suggested keeping the same hours at the 5-day conference of APPC15. Yokoyama pointed out that the Asia-Europe Physics Summit (ASEPS) time slot should be cancelled. The annual meeting of ASEPS in association with the European Physical Society (EPS) condensed-matter-division conference has been postponed and the new schedule overlaps with that of APPC15.

Weiping Liu commented that he would keep this schedule in mind on behalf of DNP and communicate with division members for their support. Normally, they have an Asian Nuclear Physics Association (ANPhA) board meeting annually in connection with an ANPhA symposium, which reports the recent progress and facility development. This year, it was planned to be held in Myanmar, but the COVID-19 situation forced them to go completely online this December. Concerning the division meeting next year, he is thinking about incorporating the ANPhA board meeting and ANPhA symposium into APPC15.

Yokoyama asked if DCMP plans to have an annual meeting this year. J.-G. Park answered that the first annual meeting will be held in December 2021, and the program committee will be chaired by Prof. Nojiri. He expects that this is a good exercise for APPC15. DCMP will not have a separate meeting in 2022 and APPC15 will be the largest meeting in 2022 for DCMP.

Division Secretary Gwak reported that he will chair the DACG online workshop this year, instead of the International Symposium on Cosmology and Particle Astrophysics (CosPA). The preparations are in progress. DACG will not have a separate meeting in 2022, and their annual meeting will be assigned inside APPC15.

Choi explained that KPS organizes two annual meetings covering all areas of physics, which are Fall and Spring Meetings. Each meeting is attended by close to 2000 participants. The Fall Meetings are held in October, and the period of APPC15 should be convenient for KPS members, without overlap.

T. Lee reported that the KPS president would have a meeting with all division representatives of KPS on 16 July 2021. Although the 12 divisions of KPS do not exactly match the AAPPS division structure, they will discuss how the KPS divisions can help to organize the parallel sessions at APPC15. The KPS divisions will recommend some Korean researchers for the scopes of APPC15.

Choi explained that the registration fee would be the same as that for APPC14—namely, a standard fee of $500 USD for postdocs and professors, and a reduced fee of $250 USD for students.

Liu gave a friendly reminder that he expects the COVID-19 situation will be very much improved in 2022, and the year will be busy with many international conferences. To avoid overlap with other conferences, we should announce the dates of APPC15 well in advance.

(6) Nomination rule for the CN Yang Award

Yokoyama reported that this year’s selection of the CN Yang Award recipients is in progress under the leadership of Prof. Kao. The award ceremony will be held in November this year. In the next year, we need to start the nomination process sooner in order to be ready for the award ceremony at APPC15 in August. We are in the process of revising the nomination rule. Presently, each division has a role in the prescreening of candidates instead of nomination. Yokoyama asked if the divisions wish to have their own nomination channels.

J.-G. Park, on behalf of DCMP, explained that he shared all information regarding the nominees with two vice chairs and the secretary-general, who formed the selection committee of DCMP. They had long discussions and the selection process worked very well, with a good exchange. He stated that DCMP does not have a particular preference regarding nomination but showed his concern over the potential for conflicts of interest when nomination and prescreening bodies were mixed. He stated that DCMP will follow a decision by the AAPPS Council.

Yokoyama stated that if we do mix nominations from divisions and those from other channels, there will be conflicts of interest. He proposed to stick to the previous rule that divisions act in the prescreening process.

E. Lee conveyed a question from a board of trustees meeting of APCTP. Since the CN Yang Award is now a joint award between AAPPS and APCTP, it might be possible for the APCTP community to have a chance to nominate. Yokoyama responded that as we discussed and concluded that divisions of AAPPS may not have a right to make nominations and their current role is to make prescreening as a part of the selection process, and no selecting body should make nominations to avoid conflicts of interest. However, APCTP has member countries/regions and they may have a right to nominate. Yokoyama asked E. Lee to collect information on how member countries/regions or membership entities of APCTP may make nominations.

Liu reported a practice employed by DNP, which seemed to work. ANPhA and AAPPS-DNP collected candidates from the nuclear physics societies in each country/region through a form of online voting. He also suggested simplifying the evaluation form, just by making a total score and single remarks.

Fu-Jen Kao agreed with Yokoyama regarding the concern of conflicts of interest. He stated that whoever is doing the selection should not make nominations, and that collecting nominations from APCTP and AAPPS seems reasonable. He emphasized that how we maintain the prestige and fairness of the CN Yang Award is an important issue. Next year, we should start asking for nominations almost as early as November to decide the awardees by July. Kao added that this year’s selection would be finished by September.

(7) Report from the Women-in-Physics Working Group of AAPPS

Y. Park gave a brief report on the activities of the Women-in-Physics (WIP) Working Group of AAPPS. She reported that the International Conference on Women in Physics (ICWIP2020) is taking place from 10 to 16 July 2021, and the AAPPS-WIP workshop will be held on 16 July. She reminded us that there should be enough female representation among organizing committee members, program committee members, plenary speakers, division-invited speakers at APPC15. She suggested having time slots for an AAPPS-WIP session, breakfast and lunch gatherings for the working group and female members at APPC15. She asked to contact her if there are any country/region members who are not participating in the working group.

(8) AAPPS Bulletin contract issues

Two files concerning the AAPPS Bulletin (AB) contract were already sent from President Yokoyama to all council members. APCTP is providing most of the funds to publish articles through cooperation with Springer Nature.

Yokoyama explained that they have made a draft of the amendment, in which three bodies are explicitly indicated, i.e., Springer Nature, AAPPS, and APCTP. The role of APCTP is to serve as a sort of sponsor. The open access fee (or APC) costs 1570 Euros per article. APCTP chooses to cover the APC for up to 20 accepted articles in the first 3 years, and up to 26 articles in the remaining 2 years. Changes in these numbers were made according to the discount provided by the publisher at the initial stage of the journal’s development. The publisher waives the APC for 10 articles in the first 3 years, and for four articles in the remaining 2 years. This means that we have secured funding to publish 30 articles every year from 2021 to 2025.

Meanwhile, there is another clause in the original publishing agreement. Clause 9.2d says that AAPPS ensures all reasonable efforts to publish “not fewer than 30 articles in 2021, not fewer than 35 articles in 2022, not fewer than 45 articles in 2023, not fewer than 55 articles in 2024, and not fewer than 70 articles in 2025.” Appendix 1–5 states the minimum requirement for the editor-in-chief. It says that the editor-in-chief shall use all reasonable efforts to ensure “not fewer than 30 articles a year.” This part is to be covered by APCTP and Springer Nature. However, the number of articles remaining, e.g., 5 articles in 2022, 15 articles in 2023, and so on, must be paid articles. Long stated that they can meet the conditions for the numbers of articles, and expressed his firm determination to achieve this. E. Lee explained that for this goal, APCTP has discussed and plans several invited review articles to be covered by APCTP. In 3 or 4 years, they will accept free submissions as well, so that not APCTP but the authors will pay the APC. (Note added: After the Council Meeting, it was confirmed that APCTP may cover these remaining articles in 2022 and 2023, and will negotiate with Springer Nature in 2023 on the numbers set for 2024 and 2025.)

In the original publication agreement, there is a clause regarding the printed version. Clause 6.3 seems to say that we are not allowed to publish peer-reviewed articles in the printed version of AB. Yokoyama expressed that this contradicts with what we are doing currently. Rajadeep Singh Rawat commented that if we do not ask permission, Springer Nature does not allow it, but they normally give permission upon request. E. Lee stated that in the previous discussion with Springer Nature, they indeed allowed APCTP to print peer-reviewed articles, though it was not written in the contract. Long suggested negotiating with Springer Nature regarding Clause 6.3 and the minimum numbers of compulsory publications. (Note added: After the Council Meeting, APCTP confirmed that Springer Nature has agreed not to apply Clause 6.3.)

Rawat wondered why a printed version of peer-reviewed articles is needed for AAPPS or APCTP as an organization. Long responded that this is based on their previous practices. All items in News and Views are now put into a single article in six issues published per year. The organization is distributing the printed version to member societies and some individuals. There may be an alternative measure for the future, but they need to stick to this approach for the time being.

Yokoyama summarized that there are still many issues. The editor-in-chief, the AAPPS president, and the president of APCTP will negotiate with Springer Nature again. Yokoyama thanked Rawat for his continued help in the interpretation of the contract.

(9) Website links to institutes in Asia Pacific

As a compensation for the discontinued section of Institutes in Asia Pacific in AB, we may create links to websites and videos of institutes in the Asia-Pacific region. Mio Murao has kindly searched for videos from some Japanese universities. Yokoyama proposed to have such a list of links on the AAPPS org website. Kurunathan Ratnavelu supported the idea. Yokoyama asked respective council members to make a list of videos and institutions in their own country/region and to ask for permission to link them in the AAPPS org website.

(10) Proposal to organize an online “Asia Pacific Physical Societies’ Forum” meeting

Until 4 years ago, considerable time was spent at each face-to-face council meeting for the reports from member societies. Yokoyama proposed having a 1-day meeting, at which each member society reports its current status. The aim is to improve mutual understanding and to discuss shared problems in the world physics community. This plan emerged through a discussion with the JSAP President, Prof. Hatano, on how we ask JSAP to continue providing their support to AAPPS. One possibility is to improve internal communication by having such a forum. All AAPPS council members are welcome, and each society will be asked to send at least one member to attend.

Yamada explained that President Hatano wishes to communicate with other member societies to understand the issues in progress and identify the problems, because the Asia-Pacific region is so important for the future progress of applied physics. Yokoyama added that the idea is to share information, not only among council members but also among members of member societies. The representatives will be selected by each society, probably from officers.

Tao Xiang agreed with the idea and suggested a half-day online meeting. Rawat expressed his support for this suggestion. The forum is planned to be held in November.

(11) Proposal to form a working group to make code of conduct of AAPPS

Yokoyama considers that it is a good time to discuss a code of conduct of AAPPS as one of the world-leading associations in physics. Article 3 of AAPPS is a sort of ethics requirement for activities. He asked if council members agree with starting by making a code of conduct for AAPPS. He proposed to form a small working group to make a draft. Ratnavelu agreed and stated that it would never be too late. Yokoyama suggested including council members from Australia, Singapore, and Malaysia, the president, and vice president, and this suggestion was approved.

(12) Next council meeting

President Yokoyama announced that a forthcoming council meeting is planned to be hosted by Prof. Zhang in Hong Kong. Saturday, 18 December was proposed as a tentative date for the hybrid-type meeting.

(13) Miscellaneous issues

Ratnavelu briefly reported on the conference on computational physics. Fifteen invited and plenary speakers were selected, and the deadline for the submission of abstracts was extended.

K.-Y. Kim reported a delay in the preparations for the formation of the division of particles and fields. They will work to accelerate the preparations, and he hopes to report on the progress at the next meeting.

Yokoyama closed the meeting by remarking that an organizing committee meeting of APPC15 will be held in a month or two, at which all council members are supposed to meet.
